# 胸腺瘤微创切除术的基本原则和标准术语的定义

**DOI:** 10.3779/j.issn.1009-3419.2014.02.05

**Published:** 2014-02-20

**Authors:** Alper Toker, Joshua Sonett, Marcin Zielinski, Federico Rea, Tomulescu Victor, Frank C. Detterbeck, 北京大学肿瘤医院 付浩

**Affiliations:** 1 Department of Thoracic Surgery, Istanbul University School of Medicine, S, ehremini, Istanbul, Turkey; 2 Department of General Thoracic Surgery, Colombia University, New York, New York; 3 Department of Thoracic Surgery, Pulmonary Hospital, Zakopane, Poland; 4 Department of Thoracic Surgery, University of Padova, Padova, Italy; 5 General Surgery and Liver Transplantation, University of Medicine and Pharmacy, Bucuresti, Romania; 6 Department of Thoracic Surgery, Yale School of Medicine, New Haven, Connecticut

根治性手术切除是胸腺瘤的主要治疗手段，手术目的在于完整切除肿瘤及胸腺组织，并且进行彻底的探查排除可能存在的非连续但可切除的病灶。通常主张胸骨劈开的整块切除，R0切除是公认的关乎远期疗效的重要因素。近年随着胸腔镜技术的不断进步，微创手术在胸腺瘤手术中的应用受到了极大地关注，但存在许多值得注意的问题，如何保护膈神经？膈神经切除后是否需要行膈肌折叠术？如何在腔镜下判断是否有上腔静脉和肺静脉的浸润？在微创手术时若切缘很近是否更容易发生胸膜播散？如果一次面对的问题太多，很有可能让微创手术的适用范围又回到从前。如果术后出现不良的结果就更难继续去探索微创技术和如何避免这些问题。多数学者仍对微创手术的结果持谨慎态度，如何确保这些结果与标准开放性手术相当是值得重视的问题。尽管微创手术缩短了住院时间并减轻了患者的疼痛，但与开放手术相比微创手术的肿瘤学优势还没能很好地被报道^[1]^。只有时间能告诉我们这些微创技术和目前的标准治疗是否相当。虽然有经验的术者采用微创手术可以切除邻近的累及器官，然而在快速拓展微创手术之前必须首先通过直接案例小心评估微创手术的预后结果。普遍认为应避免微创手术切除膈神经和大血管。由于胸腺恶性肿瘤的低发病率特点和微创手术的稀少性特点亟需有共同标准的国际合作。为此国际胸腺肿瘤协作组织（International Thymic Malignancy Interest Group, ITMIG）先由有胸腺瘤微创切除术经验的医生组成初步工作组，在回顾了目前文献中针对胸腺瘤微创切除术的定义、术语和策略后提出了初步建议，由拓展工作组进一步完善后由ITMIG工作组中的更多不同背景的专家进行讨论，最终由ITMIG的成员一致通过。内容涉及胸腺瘤微创手术策略、手术方法的基本概念、基本原则和必须遵守的特定标准等定义。以便在前瞻性的数据库采用连续和系统的方式收集数据来更恰当地评估微创手术。使不同中心之间使用这项技术应用共同的语言，便于中心之间的合作和数据资源共享。

## 1 微创手术的定义

在开放性手术过程中，大多数医生会采用整块切除，完整切除胸腺并切除胸腺颈部上极以及周围的纵隔脂肪^[2]^。微创切除手术需要同样的方式，胸腺的微创切除包括多种切口入路、暴露方式、设备和器械。如颈部切口、扩大的颈部切口、右侧或左侧/右侧+左侧/右侧+颈部/左侧+颈部/剑突下+右侧(和左侧)胸腔/颈部/剑突下电视辅助胸腔镜或机器人切口^[3]^。另一些手术还包括胸骨提拉和肋骨撑开，以及直视或者通过显示器的观察。微创胸腺切除术广义的定义指任何不进行胸骨切开术（包括部分胸骨切开术）或者带有肋骨撑开的开胸术达到肿瘤完整切除的手术方式（表 1）。这个定义是根据治疗目的来确立的，包括计划采用该治疗方式，即使最终转向开胸治疗（胸骨切开术或胸廓切开术）或者不完整切除的病例。尽管经颈部切口直视下切除胸腺具有优势，但是在切除术中一个非常有意义的部分就是应该通过显示器观察。

**1:** 微创手术原则 General principles for minimally invasive resection

1.	微创手术不涉及肋骨撑开和胸骨提拉，其目的是完全切除，最重要的部分是通过显示屏观察术野
2.	切除范围包括胸腺瘤、胸腺和纵隔脂肪组织
3.	应解剖暴露无名静脉和双侧膈神经
4.	遇到以下情况应中转开胸：侵透包膜、不完整切除、不能整块切除或组织破坏造成肿瘤暴露
5.	取标本的切口需要足够大，以避免造成标本破坏
6.	若肿瘤侵犯纵隔胸膜应探查整个胸膜腔
7.	取标本应使用标本袋
8.	应检查切除的标本，判断是否完整切除
9.	对于可疑区域应与病理医生交流。在切除过程中应注意以下问题：标本方向、需同时在标本和患者体内对常规区域标记、辨别组织破坏的区域。
注：本表得到版权所有者© 2011 by the International Association for the Study of Lung Cancer复制许可。	

总而言之，完全切除的目的不应受微创手术方式的影响，当原计划手术方式需发生改变时，开胸手术应视为标准术式，而不是微创手术失败后的并发症。

## 2 切口和暴露

切口的数量、位置和尺寸应该进行报道。还应该说明是否采用了胸骨上提。如果微创切除术是按照之前的定义进行的，即使行剑突和肋软骨切除也应归在其中，但均应予以报道（表 1）。

## 3 切除的范围

微创切除术是相对于开放手术而言的，切口和手术途径的选择不能随意改变或缩小切除范围，成功治疗的关键在于完整切除。减瘤或不完全切除的“微创”手术是不能接受的手术方式，只要微创下不能达到完全切除就应立即中转开胸，因为不断有强有力的数据证明完整切除的重要性，任何微创切除手术都不能妥协于这个原则。大宗病例报道发现即使是Ⅰ期的胸腺瘤完整切除后仍有复发的病例，因此，要求对怀疑胸腺瘤的前纵隔肿瘤的手术均应遵循恶性肿瘤的外科切除原则。有时肿瘤的外侵极其微小很难被外科医生所发现，因此术中不能仅仅剔除肿瘤，还应该包括周围的胸腺组织和脂肪组织^[4]^。如果肿瘤突入胸膜腔就有可能出现胸膜转移，外科医生应该仔细检查整个胸膜腔来寻找可能的壁层和脏层胸膜转移，如果发现转移应该进行相应切除；对于完全包绕在胸腺中的肿瘤则无需检查胸膜，应在不暴露肿瘤的情况下予以切除。对于没有重症肌无力的患者行完整的胸腺切除术即可，而对有重症肌无力的患者应该进行扩大的胸腺切除术，即在行完整胸腺切除的同时切除邻近的双侧纵隔胸膜、纵隔和心包周围的脂肪组织、以及主肺动脉窗脂肪组织。ITMIG认为除个别临床试验以外，外科微创切除术的范围不应小于上述要求。一些医生认为超根治胸腺切除术还应该包括在胸骨上提下进行颈部清扫^[5]^。

## 4 中转开胸的适应症

有医生认为，临床和影像学检查可作为微创手术适应病例的选择依据。术前或术中发现肿瘤侵犯膈神经、或喉返神经、或者侵犯大血管的征象者应中转开胸^[6]^。肿瘤侵犯膈神经、或无名静脉、或其他大脉管是微创切除术的禁忌证。须再次强调，在微创切除术中如遇任何肿瘤或外科原因致使手术有可能达不到R0切除者应毫不犹豫的中转开胸。

## 5 手术操作

### 5.1 一般方法

微创切除术应采用系统顺序的方法进行。在切除之前应该查看胸腺瘤全貌并评估是否可行微创胸腺切除术。如果肿瘤不邻近大的血管结构、膈神经、或胸骨，外科医生才可决定行微创切除术。外科医生的首要目标是切除胸腺和胸腺肿瘤，手术切除前应明确切除的界限，即左侧和右侧膈神经、胸骨的后面、心包周围、剑突下和颈部。心包周围和主肺动脉窗内的脂肪组织可完整切除也可分别切除，但胸腺瘤不应该与胸腺分开独立切除，标准的术式应该是整块切除肿瘤且在任何时候任何点尽量避免暴露肿瘤。

### 5.2 术中标本的处理

胸腺瘤的切除应该遵守“不接触”肿瘤的原则，这就意味着不应该钳夹或挤压肿瘤，因为这样会破坏瘤体并增加胸膜播散的风险，只允许轻轻地牵拉。但手术过程中发现部分组织存在潜在破坏的情况时，应该立刻标记标本及患者，应该采用清晰的标记方法，简单的钛夹标记是一种很糟糕的选择，因为术中大多数的血管也用钛夹处理。此外，破坏的区域应该和病理科医生沟通^[7]^。在微创切除术中，对于组织破坏的关注尤为重要，以便于加深组织破坏对预后影响的理解。

### 5.3 淋巴结

胸腺瘤淋巴结转移的发生率在一个日本的大宗报道中为2%，Masaoka Ⅰ期为0.4%，大约90%的转移淋巴结位于前纵隔^[8]^。微创手术中淋巴结的清扫策略应遵循ITMIG在开放手术所推荐的原则^[7]^，任何邻近的上纵隔的淋巴结或可疑的淋巴结都应该常规清扫，对分期较晚者需要广泛的淋巴结评估。

### 5.4 标本的取出

标本应该放在一个塑料袋中取出。一旦标本被完整切除，在放入塑料袋中之前应避免标本在纵隔器官表面或胸膜表面的牵拉移动，从而降低种植转移的几率。标本应该从一个足够大的切口取出，以免在拉出的过程中破坏胸腺瘤包膜或者周围组织。

### 5.5 手术记录

记录手术的细节对于分期、进一步治疗和进一步影像评估来说十分重要（表 2，表 3）。手术记录应该描述切除的标本，尤其应该描述术中所见的纵隔结构，如右侧和左侧膈神经、右侧和左侧纵隔胸膜、无名静脉、心包、上腔静脉等与肿瘤的关系和处理过程。如果在处理的过程中标本被破坏，应该在手术记录中加以标注。此外，应详细记录术中留置的止血材料的种类、特点、数量及部位，以免术后在CT扫描中与残余胸腺组织或者肿瘤复发相混淆。

**2:** 术中记录内容 Details of intraoperative report

切口数量、位置和大小，如颈部切口
是否使用胸骨提拉？
是否行剑突和肋软骨切除？
是否行肋骨撑开、胸骨提拉或肋骨切断？
是否侵犯邻近器官？若有，需一一列出。
术中暴露了哪些纵隔结构？如左/右膈神经、无名静脉、左/右纵隔胸膜、心包、上腔静脉、大血管和主肺动脉窗。
探查范围，是否左/右胸膜和心包腔可疑受侵？
列出所有切除组织，如胸腺、与肿瘤粘连的周围结构、邻近脂肪组织和淋巴结。
胸腺瘤是否整块切除？肿瘤表面是否暴露？
探查了哪些区域淋巴结？是否进行了系统性的淋巴结采样和清扫？
脂肪组织切除情况，如心包膈的、纵隔、颈部脂肪组织；是整块切除还是分别切除？
与肿瘤紧密相邻的区域是否进行了标记，术中就要同时对标本和在患者体内标记这些区域。
止血材料的使用情况，需记录型号、数量和摆放位置。
说明中转开胸的原因
注：本表得到版权所有者© 2011 by the International Association for the Study of Lung Cancer复制许可。

**3:** 推荐胸腺瘤微创手术需记录的内容 Proposed data collection for minimally invasive thymoma resection

1.	重症肌无力：是/否 如果是，对其进行MGFA分期，术前行血浆置换或IVIG
2.	切口：左/右/颈部/剑突下
3.	切口下直视，或使用显示屏，或二者均包括
4.	切除的胸腺和胸腺瘤以外的组织：心包膈脂肪组织、左/右纵隔胸膜、前纵隔淋巴结和颈部脂肪组织
5.	切除、暴露和探查的结构：无名静脉、左/右膈神经和左/右纵隔胸膜
6.	取出标本切口的长度
7.	切除情况：单纯胸腺瘤切除、联合胸腺整块切除、联合脂肪和胸腺整块切除、联合颈部脂肪整块切除和联合上述结构的非整块切除
8.	淋巴结切除情况：未切除；前纵隔、气管旁、隆突下、颈部淋巴结切除；系统性采样或淋巴结清扫
9.	胸膜腔探查：左/右
10.	Masaoka-Koga分期
11.	组织学特征
12.	并发症
13.	手术持续时间
14.	ICU住院时间
15.	术后住院时间
16.	中转开胸：是/否
IVIG：静脉输注丙种球蛋白；MGFA：美国重症肌无力功能分期。 注：本表得到版权所有者© 2011 by the International Association for the Study of Lung Cancer复制许可。	

### 5.6 切除标本的处理

对微创手术切除标本进行合理评估极其困难，但该步骤对患者预后的评估非常重要，病理组织学结果是预后和术后治疗的关键决定因素。术中操作范围的限制和标本取出过程的困难会造成标本从塑料袋中被取出时出现胸腺瘤的包膜与周围脂肪组织分离的情况。外科医生和病理医生之间在肿瘤的大体标本上的交流评估以确定切除的完整性十分必要。

当对侵袭性肿瘤进行完整切除时，冰冻切片有助于外科医生去评估切缘的充分性，然而冰冻切片的解释十分困难。外科医生应该明白胸腺瘤冰冻切片有很高的假阳性和假阴性率^[7]^，因此，术中肉眼仔细评估切除标本对判断切缘十分关键。外科医生应该像开放手术一样对标本进行定位^[7]^。在开放手术中邻近心包、无名静脉、以及右侧和左侧纵隔胸膜和上腔静脉的标本表面推荐常规标记。采用纵隔板或图解表示邻近结构的定位和性质是外科医生和病理科医生之间沟通简单有效的方式^[7]^。而外科医生和病理医生之间（或者是手术助手和准备大体标本的病理科工作人员之间）的面对面交流也十分必要。交流的内容包括有任何潜在近切缘可疑切缘不够的区域和术中组织破坏区域的识别，这些问题在术中很容易被解决，常规手术录像有助于帮助病理医生理解他们关注的部分。标本的处理和病理结果报告应该跟开放手术一样^[7]^。切除胸腺瘤周围脂肪组织对于伴有重症肌无力的患者来说是另一个重要的问题。对于这些患者胸腺外组织的病理分析有助于研究针对重症肌无力治疗的研究数据。对胸腺上皮肿瘤进行病理评估时应该采用表格式报告单^[9]^。本文和其他关于开放手术标本处理的文章的主要目的在于提供一个框架供大家参考使用^[7]^。

总之，胸腺的微创手术要求与开放手术有同样的切除质量与评价体系。
